# Voice analysis results in individuals with Alzheimer's disease: How do age and cognitive status affect voice parameters?

**DOI:** 10.1002/brb3.3271

**Published:** 2023-10-04

**Authors:** Mümüne Merve Parlak, Güleser Saylam, Mehmet Ali Babademez, Özlem Bizpınar Munis, Sibel Alicura Tokgöz

**Affiliations:** ^1^ Department of Speech and Language Therapy, Faculty of Health Sciences Ankara Yıldırım Beyazıt University Ankara Turkey; ^2^ Department of Otolaryngology Etlik City Hospital Ankara Turkey; ^3^ Department of Otolaryngology Ankara Bilkent City Hospital Ankara Turkey; ^4^ Department of Neurology Etlik City Hospital Ankara Turkey

**Keywords:** Alzheimer's disease, cognitive, dementia, voice analysis, voice disorders, voice therapy

## Abstract

**Background:**

Reports of acoustic changes in the voice in individuals with Alzheimer's disease (AD) and the relationship of acoustic changes with age and cognitive status are still limited.

**Objective:**

This study aims to determine the changes in voice analysis results in AD, as well as the effects of age and cognitive status on voice parameters.

**Methods:**

The study included 47 (AD: 30; healthy: 17) women with a mean age of 76.13 years. The acoustic voice parameters mean fundamental frequency (*F*0), relative average perturbation (RAP), jitter percent (Jitt), shimmer percent (Shim), and noise‐to‐harmonic ratio were detected. The mini‐mental state examination (MMSE) was utilized.

**Results:**

*F*0, Shim, Jitt, and RAP values were found to be statistically significantly higher in individuals with AD compared to healthy individuals. There was a significant negative correlation between MMSE and *F*0, Jitt, RAP and Shim, and the MMSE score had a significant negative effect on *F*0, Jitt, and RAP (*p* < .05).

**Conclusion:**

Cognitive status was discovered to significantly impact the voice, with fundamental frequency and frequency and amplitude perturbations increasing as cognitive level decreases. In order to contribute to the therapy process for voice disorders, cognitive functions can be focused on in addition to voice therapy.

## Graphical abstract

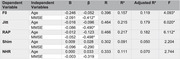

## 1 INTRODUCTION

Normal phonation is a complex end product that requires appropriate neuromotor, respiratory, and laryngeal control. Disease processes affecting any or all systems tend to disrupt normal phonation. Studies have shown that various neurodegenerative or chronic neurological diseases show differences in the acoustic and auditory perceptual analysis of voice (Mayo‐Yáñez & Cabo‐Varela, 2020). Alzheimer's disease (AD) is a progressive neurodegenerative disease in which cognitive functions are affected, with an increase in frequency with age (Munis et al., 2021; Parlak, Köse et al., 2023; Parlak, Tokgöz et al., 2022). As AD progresses, sensory and motor performance deteriorate (Parlak, Altan et al., 2022; Parlak, Babademez et al., 2022). With advancing age and the progression of AD, there may be single or multiple causes involved in voice dysfunction (Mayo‐Yáñez & Cabo‐Varela, 2020).

Bowing, persistent air escape, poor voice production, and hyperfunctional poor breath support have been observed in some studies in individuals with AD. However, there are very few studies in the literature that have analyzed the voice of individuals with AD, and these studies have focused on speech analysis rather than voice quality. These studies recorded the voices of patients with suspected AD performing several short cognitive vocal tasks and analyzed the speech sounds using speech signal processing techniques. According to the results of these analyses, they aimed to determine the classification of individuals as normal, having AD, or having mild cognitive impairment (König et al., 2015). However, the impact of this neurodegenerative disease on voice has been overlooked and not studied in detail due to the focus on cognitive impairments brought on by the disease, whereas other aspects of communication such as voice are largely under‐researched in this population. Reports of acoustic changes in the voice in individuals with AD and the relationship of acoustic changes with age and cognitive status are still limited. In addition, the effect of cognitive status on voice in healthy individuals is a relatively current research area. If the effect of cognitive level can be demonstrated in both healthy and AD individuals, the voice parameters that can be changed by focusing on cognitive functions in healthy individuals with voice disorders can be determined, and it can be demonstrated that voice disorders can be intervened with cognitive therapies in addition to voice therapy. In addition, voice parameters associated with the determination of the relationship between cognitive status and voice analyses can be used as biomarkers for screening and identification of individuals with cognitive impairments and for the prognosis and staging of AD. Therefore, in this study, it was aimed to determine the changes in voice analysis results according to the stages in individuals with AD, examine the effect of AD on voice parameters by comparing them with the voice analysis results of healthy elderly individuals, and determine how age and cognitive status affect voice parameters.

## 2 MATERIALS AND METHODS

The study was conducted at the Dementia Outpatient Clinic of the Neurology Clinic and the Voice Outpatient Clinic of the Otorhinolaryngology Clinic of the University of Health Sciences Dışkapı Yıldırım Beyazıt Training and Research Hospital. The Ethics committee approved the study was obtained from Dışkapı Yıldırım Beyazıt Training and Research Hospital with permission number 66/14.

### 2.1 Participants

For both the AD and healthy control groups, having an upper respiratory tract infection during the evaluation, not being able to cooperate with the test during the applications, receiving treatment or having surgery for any voice problem, having oropharyngeal surgery, having laryngeal and craniofacial medical histories, having a smoking history of more than 3 years, and having additional neurological diseases such as Parkinson's disease or multiple sclerosis were determined to be exclusion criteria.

For the Alzheimer group, being followed up in the neurology clinic and previously diagnosed with AD using Diagnostic and Statistical Manual for Mental Disorders, fifth edition and National Institute on Aging and Alzheimer's Association criteria and being able to phonate /a/ vocal for at least 3 s were determined as inclusion criteria. The inclusion criteria for the control group were not having any vocal disease and not having any pathological or physiological problem, neurological disease, and lung disease that may disrupt the formation of the voice.

As different physiological mechanisms are involved in voice disorders seen in female and male with aging, only women were included in our study (Rojas et al., 2020). In this way, it was aimed at ensuring homogeneity in voice parameters. The study was carried out on 47 females, consisting of 30 individuals with AD and 17 healthy females who met the inclusion and exclusion criteria.

### 2.2 Voice assessment

In the insulated voice analysis room, the participants were asked to phonate the vocal /a/ a for 3 s after taking a deep breath while sitting in a chair or wheelchair. Shure SM48 microphone was used during the recording. During voice recordings, the distance between the patient and the microphone was adjusted to be 4 in. (15 cm). The mean fundamental frequency (*F*0), relative average perturbation (RAP), jitter percent (Jitt), shimmer percent (Shim), and noise‐to‐harmonic ratio (NHR) acoustic voice parameters were determined at the Computerized Speech Laboratory programs using the multidimensional voice analysis program Multi Dimensional Voice Parameters. The use of these voice parameters provides general information about frequency, frequency and amplitude perturbations, and noise in voice (Kilic, 2010).

*F*0 indicates the number of vibrations of the vocal folds in 1 s. Jitt indicates the frequency differences between successive periods. Percent jitter was used in the study. The reason for this is to eliminate the drawback of absolute jitter depending on the fundamental frequency. This value is calculated by dividing the absolute jitter by the average period. RAP: A very short periodic change in the timbre of a voice sample, found by calculating the difference between the average of three consecutive periods and the period in the middle of these three periods. It is a calculation method used to ensure that fundamental frequency changes such as voluntary or involuntary vocal tremor or the inability to keep one's voice at the same pitch do not affect jitter values. RAP and Jitt provide a measurement of frequency irregularity in vocal folds. Shim shows the amplitude differences between successive periods. It is a measure of the change in the amplitude of the voice. Percent shimmer was used in the study. It is calculated by dividing the average of the absolute value of the difference in amplitude between each period and the following period by the average period amplitude. It provides measurements of irregularities in amplitude. NHR is the ratio of the noise energy to the total energy of the fundamental frequency and its multiples, the harmonics. High values indicate that the noise ratio in the voice is high. It is the numerical equivalent of the extra noise heard in the voice (Kilic, 2010). These parameters are basic voice parameters and are used in many voice studies for evaluation and therapeutic research purposes (Munoz et al., 2003; Onen et al., 2021; Saltürk et al., 2019; Tunç‐Songur et al., 2023).

### 2.3 Cognitive assessment

The general cognitive assessment of the individuals who met the inclusion and exclusion criteria was performed with the standardized mini‐mental state examination (MMSE). It is a cognitive screening test frequently used in studies on AD (Munis & Parlak, 2023; Parlak, Bizbinar et al., 2023; Parlak, Köse et al., 2023). MMSE was scored over a total of 30 points. The Turkish validity and reliability study of the test developed by Folstein et al. (1975) was conducted by Güngen et al. (2002).

The Clinical Dementia Rating Scale (CDR) was used to determine the stage of AD. With the CDR, both the stage of the disease and changes in the clinical status of patients over time can be monitored. The scores are, respectively, normal old age (0 points), mild cognitive impairment (0.5 points), mild dementia (1 point), moderate dementia (2 points), and advanced dementia (3 points) (Hughes et al., 1982). In the literature, the CDR scale is generally used to stage AD, and the disease is analyzed in three stages: mild (early), moderate, and advanced (severe/late) AD (Elif et al., 2017; Gürvit, 2004). In our study, individuals with AD were classified into three stages according to their CDR scores: mild with a score of 1, moderate with a score of 2, and advanced with a score of 3.

### 2.4 Data analysis

The data to be obtained were analyzed with the SPSS 26 program. For descriptive analyses, categorical variables were evaluated as number and percentage, normally distributed numerical variables were evaluated as mean and standard deviation, and non‐normally distributed numerical variables were evaluated as median (minimum–maximum). Whether the data related to the variables were normally distributed or not was analyzed with kurtosis and skewness values. As a result of the normality distributions of the obtained data, the ANOVA or Kruskal–Wallis *H* test was used for comparisons with three or more groups, and the Mann–Whitney *U* test was used for comparisons with two groups. Pearson's or Spearman's test was used to analyze the relationship between numerical variables according to their normal distribution. The effect of independent variables on the dependent variable was analyzed by regression analysis. Age and MMSE score were used as independent variables in the regression analyses, whereas voice parameters were used as dependent variables. In the analyses, *p* < .05 value was considered statistically significant.

## 3 RESULTS

The mean age of individuals with AD was 76.13 ± 7.890 years, whereas the mean age of healthy individuals was 68.31 ± 6.052 years, which was statistically significantly higher. The mean MMSE was 13.87 ± 5.258 in individuals with AD and 26.88 ± 2.062 in the control group (Table 1).

**TABLE 1:** Comparison of parameters between healthy and Alzheimer's disease (AD) individuals.

	Individuals with AD (*N*: 30) Mean ± SD	Healthy individuals (*N*: 17) Mean ± SD	*Z*	*p*
Age	76.13 ± 7.890	68.31 ± 6.052	−3.295	**.001**
MMSE	13.87 ± 5.258	26.88 ± 2.062	−5.499	**.000**
*F*0	228.58 ± 41.211	205.82 ± 29.417	−1.931	**.035**
Jitt	1.95 ± 1.449	0.86 ± 0.681	−2.941	**.003**
RAP	1.17 ± 0.858	0.51 ± 0.421	−2.896	**.004**
Shim	5.26 ± 2.631	3.69 ± 1.968	−2.043	**.041**
NHR	0.16 ± 0.068	0.13 ± 0.039	−1.595	.111

*Note*: The bold values are considered statistically significant for *p* < .05.

Abbreviations: *F*0, fundamental frequency; Jitt, jitter percent; NHR, noise‐to‐harmonic ratio; RAP, relative average perturbation; SD: standard deviation; Shim: shimmer percent.

The mean *F*0 228.58 ± 41.211, Jitt 1.95 ± 1.449, RAP 1.17 ± 0.858, and Shim 5.26 ± 2.631 were calculated for women with AD. In healthy women, *F*0 was 205.82 ± 29.417, Jitt 0.86 ± 0.681, RAP 0.51 ± 0.421, and Shim 3.69 ± 1.968. *F*0, Shim, Jitt, and RAP values were found to be statistically significantly higher in individuals with AD compared to healthy individuals (*p* < .05). The NHR did not show a statistically significant difference between individuals with AD and healthy individuals (Table 1).

*F*0, Jita, RAP, Shim, and Jitt values were found to be statistically significantly different between AD stages and healthy individuals. The NHR value did not show a statistically significant difference between AD stages (Table 2).

**TABLE 2:** Comparison of parameters in different stages of Alzheimer's disease (AD) and healthy individuals.

	Advanced stage (*N*: 8) Mean ± SD	Moderate stage (*N*: 12) Mean ± SD	Mild stage (*N*: 10) Mean ± SD	Healthy (*N*: 17) Mean ± SD	*X* ^2^	*p*
Age	75.60 ± 9.788	76.12 ± 8.717	76.44 ± 5.769	68.31 ± 6.052	10.877	**.012**
MMSE	7.20 ± 3.768	12.76 ± 2.905	19.67 ± 3.391	26.88 ± 2.062	38.791	**.000**
*F*0	263.63 ± 44.305	229.20 ± 37.759	207.95 ± 35.896	205.82 ± 29.417	8.763	**.033**
RAP	2.03 ± 0.665	0.87 ± 0.622	1.26 ± 1.048	0.51 ± 0.421	14.707	**.002**
Shim	6.41 ± 2.951	4.41 ± 2.343	6.23 ± 2.683	3.69 ± 1.968	7.972	**.047**
NHR	0.23 ± 0.120	0.15 ± 0.054	0.15 ± 0.031	0.13 ± 0.039	4.997	.172
Jitt	3.43 ± 1.094	1.45 ± 1.025	2.07 ± 1.806	0.86 ± 0.681	14.883	**.002**

*Note*: The bold values are considered statistically significant for *p* < .05.

Abbreviations: *F*0, fundamental frequency; Jitt, jitter percent; MMSE, standardized mini–mental state examination; NHR, noise‐to‐harmonic ratio; RAP, relative average perturbation; SD, standard deviation; Shim, shimmer percent. in bold .

In the correlation analysis, a significant negative correlation (*p* < .05) was found between age and MMSE (*r* = −.371), between MMSE and *F*0 (*r* = −.393), Jita (*r* = −.430), Jitt (*r* = −.505), RAP (*r* = −.494), and Shim (*r* = −.301) (Table 3).

**TABLE 3:** Correlation analysis results between age, mini‐mental state examination (MMSE), and voice parameters.

		Age	MMSE
Age	*r*	1.000	−.371^a^
	*p*	–	**.010**
*F*0	*r*	.101^a^	−.393^a^
	*p*	.499	**.003**
Jitt	*r*	.106	−.505
	*p*	.478	**.000**
RAP	*r*	.076	−.494
	*p*	.612	**.000**
Shim	*r*	.135^a^	−.301^a^
	*p*	.364	**.040**
NHR	*r*	.153	−.227
	*p*	.306	.124

*Note*: The bold values are considered statistically significant for *p* < .05.

Abbreviations: *F*0, fundamental frequency; Jitt, jitter percent; NHR, noise‐to‐harmonic ratio; RAP, relative average perturbation; Shim, shimmer percent.

^a^ Pearson's correlation test.

Table 4 shows the results of the regression analyses that were done to see what effect age and MMSE score had on the voice parameters. Age and MMSE score explained 11.9% of the variance in parameter *F*0, and the model was statistically significant (*F* = 4.093; *p* < .05). MMSE score was found to have a significant negative effect on the *F*0 parameter (*β* = −.412; *p* < .05). Age and MMSE score explained 17.9% of the variance in the Jitt parameter, and the model was statistically significant (*F* = 6.020; *p* < .05), MMSE score had a significant negative effect on the Jitt parameter (*β* = −.490; *p* < .05). It was found that age and MMSE score explained 18.2% of the variance in the RAP parameter, and the model was statistically significant (*F* = 6.112; *p* < .05), and MMSE score had a significant negative effect on the RAP parameter (*β* = −.498; *p* < .05).

**TABLE 4:** Regression analysis results.

Dependent variable	Independent variables	*B*	*β*	*R*	*R* ^2^	Adjusted *R* ^2^	*F*
*F*0	Age	−.246	−.052	.396	.157	.119	4.093^a^
	MMSE	−2.091	−.412^a^				
Jitt	Age	−.016	−.096	.464	.215	.179	6.020^a^
	MMSE	−.086	−.490^a^				
RAP	Age	−.012	−.123	.466	.217	.182	6.112^a^
	MMSE	−.052	−.498^a^				
Shim	Age	.009	.028	.302	.091	.050	2.204
	MMSE	−.096	−.290				
NHR	Age	.000	.033	.333	.111	.070	2.744
	MMSE	−.003	−.319				

Abbreviations: *F*0, fundamental frequency; Jitt, jitter percent; NHR, noise‐to‐harmonic ratio; RAP, relative average perturbation; SD: standard deviation; Shim, shimmer percent.

^a^ *p* < .05 considered statistically significant.

## 4 DISCUSSION

In the literature, there are studies examining the relationship between aging and voice and determining biomarkers with speech analysis in AD. However, in our study, with a multidisciplinary team of speech‐language pathology, otolaryngology, and neurology, we examined the difference of the voice, which is important for communication, from typical aging in AD, and investigated the effect of age and cognitive status on basic vocal parameters. The most important feature of this study that distinguishes it from other studies was the inclusion of all AD patients, including advanced stage, and the recording of a simple /a/ phonation task. This facilitated the completion of the recording especially in advanced patients. This is because many advanced stage patients may have intense coordination problems and lose the ability to speak. However, they were able to phonate /a/ for 3 s with the consecutive repetition of the other person. In addition, the results of our study have multiple implications in line with its objectives. Our results were analyzed and interpreted in both voice disorders and cognitive framework. Other studies for biomarkers in AD have focused only on cognitive features. To our knowledge, there is no study that examines both voice disorders and cognitive function in AD and examines their relationship and effect levels. In addition, it is thought that our study may be a model study for voice and cognitive features in healthy elderly individuals.

It is thought that the neuropathology of AD may lead to impaired voice production and that these individuals may have a unique profile of voice characteristics. In our study, it was observed that all voice parameters except NHR differed between AD and healthy women and between AD stages and increased with increasing severity of AD. The increase in Shim values indicates irregular glottic closure and difficulty in adjusting the stability of the loudness. The increase in Jitt and RAP values indicates involuntary tremor and difficulty in adjusting the stability of the pitch. This result showed that an individual with AD may have worse voice quality than an older adult with typical cognition. Sarcopenia of the thyroarytenoid muscles is reported to be seen in typical aging (Parlak, Köse et al., 2023), and the level of sarcopenia increases in AD. This may cause altered laryngeal muscle tone in AD and may provide a rationale for possible voice differences in individuals with AD. Although there are very few studies in the literature on voice analysis in individuals with AD, in these studies, *F*0 was found to be higher than expected in individuals with AD and higher than healthy individuals (Farrús & Codina‐Filbà, 2020; Meilán et al., 2014). Our findings supported those of other studies in the literature. This result is important in terms of the effect of AD on *F*0 and may be due to the effect of the neurodegenerative state on altered laryngeal muscle tone in AD. AD is characterized by cortical deterioration and may cause atrophy of the internal thyroarytenoid due to the cortical effects of AD. This may have caused thinner vocal folds and a higher *F*0 by decreasing the vocal fold mass (Çiyiltepe & Şenkal, 2017).

The *F*0, Jitt, Shim, and RAP values of healthy women in our study are very similar to the findings of Goy et al. (2013) with a similar average age. These results also showed that the results of voice analyses were compatible with women in a similar age range in the absence of a neurological condition that may affect the voice, such as AD. In addition, this result showed that AD had an effect on both frequency and amplitude perturbations. Perturbation measurements are called acoustic parameters indicating irregularity. High jitter values reflect the instability of vocal fold vibration, high RAP values reflect the fundamental frequency change due to the inability to keep the voice at the same pitch, and high shimmer values reflect the irregularity in glottic closure. These findings may be explained by the fact that, in addition to normal aging, the presence of AD may affect vocal fold vibrations; the effect on vibration may cause the shimmer to change or increase the amplitude by affecting the subglottal pressure in the vocal tract (Johns et al., 2011), and peripheral vocal fatigue may increase the pitch and amplitude (Simpson & Rosen, 2008).

Midi et al. (2011) compared the voice analysis findings in healthy individuals with those of individuals with MCI (mild cognitive impairment), mild AD, and moderate AD and found no significant difference between the groups in *F*0, Jitt, and Shim values. They attributed this to the homogeneity of the age variable between the groups (Midi et al., 2011). In our study, although we thought that age variation between AD and healthy subjects might have an effect on the results, our finding that there was no significant effect in the regression results refuted this thought. The fact that we included especially advanced‐stage AD in our study and the mean MMSE of the patients was lower compared to this study may be the reason for the difference in our results. In one study, it was found that shimmer values differed significantly between MCI and healthy individuals (Themistocleous et al., 2020); in another study, it was found that shimmer values differed statistically significantly between healthy and AD individuals (Meilán et al., 2014). In our study, however, both Shim and Jitt values differed between AD and healthy subjects, as well as between AD stages. The participants in our study had a wider range of MMSE scores, which may have contributed to this result. Furthermore, the participants' wide range of MMSE scores, ranging from 5 to 30, may have made the effect of AD on cognitive status and the effect of cognitive status on voice more apparent.

In studies looking at voice change with age in geriatric individuals, it has been observed that *F*0 decreases with increasing age in women, and it has been reported that a slight decrease (10−15 Hz) occurs in *F*0 in women until menopause and then remains almost constant (Sataloff & Linville, 2006). In our study, all women were past menopause, and it was observed that increasing age had a negative effect on *F*0, but it was statistically insignificant. In other studies, *F*0 values were compared in different age ranges to look at the effect of age, whereas in this study, this result was obtained by regression analysis. The effect of additional factors may have caused this result as a collective analysis was performed with individuals with AD.

In our study, according to the regression analysis, the decrease in MMSE affected the increase in *F*0 value. The dose of medication taken by individuals with AD changes as the stage progresses. Patients with AD are usually polymedicated with drugs such as diazide, theophylline, and steroid inhalers, which often cause mucosal drying. Desiccated laryngeal mucosa is expected to decrease *F*0 (Rojas et al., 2020), but *F*0 was increased. This may be due to muscle atrophy being more intense than mucosal desiccation. Samlan et al. (2018) found that cognitive impairment in women affected the voice and that physical frailty was associated with greater voice impairment. Therefore, increasing frailty levels in individuals with AD toward the advanced stage may also affect the results, but frailty was not measured in our study. A systematic review identified Jitt, RAP, and ShimdB values as voice parameters affected by aging (Rojas et al., 2020). In our study, it was observed that MMSE scores had a greater effect on Jitt, Shim, and RAP values than age, and that decreasing MMSE total scores increased these voice parameters. Therefore, the main factor affecting this result was the presence of AD, that is, cognitive impairment. Although the mean age of the AD group was higher than that of healthy individuals, both groups were over 65 years old. This showed that the effect of age decreases after the age of 65, and the effect of cognitive impairment is at the forefront. Lin et al. (2020) found both jitter and shimmer to be significantly related to cognitive level. Mahon and Lahman (2022) found that the increase in jitter was statistically significant for cognitive status. The fact that different results were obtained from some other studies in the literature comparing voice with cognition and voice with age may have resulted from the fact that the cognitive assessment scales and voice assessment protocols used were different. However, although there are different voice parameters in all studies, the effect of cognitive status on voice cannot be trivialized.

In our study, NHR did not show a significant difference between AD stages or between healthy individuals and AD, as Meilán et al. (2014). In addition, NHR was found to be a traditional voice parameter that was not affected by age or cognitive status. The fact that the NHRs were the same between the groups suggested that the voice quality was similar in terms of the environment and that there was no effect of ambient noise on the effect of other voice parameters. Pessin et al. (2017) found that when they compared the age range of 60–75 years with the age range of 76 years and older, they did not observe a relationship with age in NHR values. When the results of this study are considered, it can be assumed that NHR is not affected by age and cognitive status at a similar age level in general, especially in individuals over 60 years of age, if the ambient noise level is the same or if there is no factor that creates noise at the vocal fold level.

### 4.1 Limitations

In this study, only the participants' voices were analyzed; the perceptual evaluation of voice was not examined. In addition, the study was conducted only with female participants. Therefore, information about the relationship between voice analysis results and cognitive status in males could not be obtained. Although no relationship was found between age and voice parameters, as shown in Table 3, the age difference between individuals with AD and healthy individuals constitutes another limitation of the study. Another limitation of the study is that, although only people with a diagnosis of AD were included, a proportion of the AD cohort may have dementia caused by other etiologies.

## 5 CONCLUSION

In this study, it was determined that the values of basic voice parameters except NHR increased as the stage of AD progressed and that the values were higher in individuals with AD than in healthy individuals. In contrast to age, cognitive status was found to have a significant effect on voice. As the cognitive level decreased, it was determined that there was an increase in fundamental frequency and frequency and amplitude perturbations. It was observed that cognitive impairment had a significant negative effect on basic voice parameters such as *F*0, Jitt, and RAP. Therefore, RAP and jitter values may be indicators of cognitive impairment in the absence of additional organic, functional, or neurological conditions that may affect the voice other than AD. In addition, increases in these voice parameters have a negative effect on voice, and voice plays an important role in human communication. Therefore, by slowing down the progression of AD, that is, by reducing the rate of cognitive impairment, the incidence of voice disorders that may occur in individuals with AD can be reduced. In order to contribute to the therapy process for voice disorders, cognitive functions can be focused on in addition to voice therapy.

## AUTHOR CONTRIBUTIONS

**Mümüne Merve Parlak**: Conceptualization; investigation; funding acquisition; writing—original draft; methodology; validation; visualization; writing—review and editing; data curation; resources. **Güleser Saylam**: Methodology; resources; project administration; investigation; conceptualization. **Mehmet Ali Babademez**: Conceptualization; methodology; resources. **Özlem Bizpınar Munis**: Methodology; writing—review and editing; resources; conceptualization. **Sibel Alicura Tokgöz**: Conceptualization; investigation; writing—review and editing.

## CONFLICT OF INTEREST STATEMENT

The authors have no conflicts of interest to declare.

### PEER REVIEW

The peer review history for this article is available at https://publons.com/publon/10.1002/brb3.3271.

## Data Availability

All data generated or analyzed during this study are included in this article. Further enquiries can be directed to the corresponding author.
